# UNREPRESENTED TO FLUSH-WITH-FAMILY: THE ROLE OF FAMILY STRUCTURE ON DEMENTIA CARE BY RURALITY

**DOI:** 10.1093/geroni/igae098.2220

**Published:** 2024-12-31

**Authors:** Rebecca Utz, Eli Iacob, Mike Hollingshaus, Erin Bouldin, Thomas Cudjoe, Katherine Miller, Tim Farrell, Katherine Ornstein

**Affiliations:** University of Utah, Salt Lake City, Utah, United States; University of Utah, Salt Lake City, Utah, United States; University of Utah, Salt Lake City, Utah, United States; University of Utah, Salt Lake City, Utah, United States; Johns Hopkins University School of Medicine, Baltimore, Maryland, United States; Johns Hopkins University, Baltimore, Maryland, United States; University of Utah, Salt Lake City, Utah, United States; Johns Hopkins University, Baltimore, Maryland, United States

## Abstract

U.S. policy and clinical practice increasingly recognize the value and essential role that family caregivers play in planning and delivering long-term care, especially in the context of dementia where cognitive decline leads to practical and legal needs to rely on others (most often family) to serve as caregivers and surrogate decision makers. Given the preference of older adults to age-in-place and that younger generations commonly move away from rural areas, access to family support may characteristically differ across those living in urban versus rural areas. This study uses the Utah Caregiving Population Study (C-PopS), a unique population-based data resource that links administrative and health records with genealogical data, to describe the size and structure of first-degree family (FDF) members (spouse, child, sibling) associated with a cohort of persons aged 65+ who died with dementia in Utah, stratified by rural (n=11,105) vs. urban (n=38,099) geographic small-areas. Compared to those in urban areas, persons with dementia in rural settings were more likely to be married (39.3% versus 35.2%), but less likely to have adult children in the state (61.9% versus 67.9%). About one in five rural older adults with dementia (20.1%) were unrepresented (defined as no FDF present in the state), compared to about 17% of those in urban areas. Understanding rural-urban differences in how family structure characteristics are associated with distinct patterns of dementia care, including use of hospice and care transitions at end-of-life, will help inform health policy and future interventions targeting the unique needs of this vulnerable population.

